# Leaves and Tree Rings as Biomonitoring Archives of Atmospheric Mercury Deposition: An Ecophysiological Perspective

**DOI:** 10.3390/plants14091275

**Published:** 2025-04-22

**Authors:** Fabrizio Monaci, Davide Baroni

**Affiliations:** 1 Department of Life Science, University of Siena, Via A. Mattioli 4, 53100 Siena, Italy; 2 National Biodiversity Future Center (NBFC), 90133 Palermo, Italy; 3 Department of Environmental, Earth and Physical Sciences, University of Siena, Via A. Mattioli 4, 53100 Siena, Italy; davide.baroni@unisi.it

**Keywords:** tree rings, foliar uptake, biomonitoring, dendrochronology, ecophysiology, atmospheric mercury, stable isotope analysis

## Abstract

Trees mediate critical biogeochemical cycles involving nutrients, pollutants, water, and energy at the interface between terrestrial biosphere and atmosphere. Forest ecosystems significantly influence the global cycling of mercury (Hg), serving as important sinks and potential sources of re-emission through various biotic and abiotic processes. Anthropogenic Hg emissions, predominantly from industrial activities, mining, and fossil fuel combustion, have substantially altered the natural Hg cycle, intensifying ecotoxicological concerns and establishing forests as primary routes for atmospheric Hg deposition into terrestrial reservoirs. This perturbation profoundly affects global atmospheric Hg concentrations, residence times, and spatial distribution patterns. While early investigations focused on forest stands near heavily polluted areas, contemporary research has expanded to diverse ecosystems, revealing that trees provide tissues that function as temporal archives for atmospheric-terrestrial Hg exchange. Leaves capture high-resolution records of contemporary Hg dynamics at sub-annual timescales, whereas annual growth rings preserve multi-decadal chronologies of historical atmospheric exposure. Incorporating this dual temporal perspective is crucial for analysing Hg deposition trends and assessing the efficacy of environmental policies designed to control and mitigate Hg pollution. This review critically evaluates recent developments concerning the ecophysiological determinants of Hg accumulation in trees, highlighting how combined foliar and dendrochemical analytical methods strengthen our mechanistic understanding of vegetation-atmosphere Hg exchange. To enhance biomonitoring approaches, we emphasised the need for methodological standardisation, deeper integration of ecophysiological variables, and consideration of climate change implications as priority research areas. Furthermore, integrating Hg measurements with functional markers (δ^13^C and δ^18^O) and Hg isotope analyses strengthens the capacity to differentiate between physiological and environmental influences on Hg accumulation, thereby refining the mechanistic framework underlying effective tree-based Hg biomonitoring.

## 1. Introduction: The Role of Trees in Atmospheric-Terrestrial Mercury Exchange Dynamics

Trees form a critical link between the terrestrial biosphere and atmosphere, acting as dynamic regulators of biogeochemical cycles [1,2]. Through extensive root networks, woody biomass, and foliage, trees facilitate the movement and transformation of nutrients and elements while governing water and energy fluxes across environmental compartments [3,4]. Although much research has focused on the cycling of essential bioelements, recent studies have highlighted the pivotal role of trees and forests in global mercury (Hg) dynamics [5,6].

Mercury is a toxic, bioaccumulative pollutant that circulates globally through atmospheric transport pathways. Anthropogenic Hg emissions, predominantly from industrial activities, mining, and fossil fuel combustion, have substantially altered the natural Hg cycle, posing significant ecological and human health risks [7,8]. The metal’s toxicological significance derives principally from its capacity, particularly in its neurotoxic methylated form (MeHg), to penetrate both blood-brain and placental barriers, thereby inducing severe neurological and developmental impairments, especially in foetuses and young children. These neurophysiological disruptions manifest in quantifiable IQ deficits and elevated cardiovascular risks, imposing substantial global health and economic burdens [9].

The troposphere contains Hg primarily as gaseous elemental mercury (GEM; Hg^0^), along with smaller proportions of gaseous oxidised mercury (GOM; Hg^2+^) and particulate-bound mercury (PBM). GEM comprises over 95% of total atmospheric Hg and has an atmospheric residence time of 0.5–1.0 years, allowing it to travel great distances from emission sources [10]. Current atmospheric Hg concentrations result from anthropogenic emissions, natural sources, and the re-release of legacy Hg, generating a persistent, multi-compartmental cycle [11,12]. Ambient GEM concentrations typically range from approximately 1.0 to 2.0 ng m^−3^ over continental regions in the Northern Hemisphere. Natural sources include volcanic eruptions, geothermal vents, wildfires, and volatilisation from water and soil, whereas anthropogenic releases stem primarily from fossil fuel combustion, industrial processes, and gold mining [13].

Forest ecosystems play a significant role in atmospheric-terrestrial Hg exchange. The foliar assimilation of GEM accounts for 60–90% of the total Hg deposition on land [5,7]. This process starts with stomatal uptake, followed by intracellular oxidation to Hg^2+^, chelation with thiol-rich compounds, and eventual sequestration as β-HgS nanoparticles [14,15]. Over time, much of this foliar-bound Hg is transferred to forest soils through litterfall and throughfall, where it may accumulate in organic horizons or be converted to MeHg [16,17]. Although this sequestration renders forests powerful Hg sinks, they can also act as secondary sources during disturbances such as deforestation, wildfires, and land-use change [18,19].

From a biomonitoring perspective, trees offer dual temporal perspectives on atmospheric Hg trends. During the growing season, leaves integrate ongoing GEM uptake, providing time-dependent exposure data over periods of weeks to months at a high spatial resolution [20,21]. At the same time, tree rings may record multi-decade atmospheric Hg fluctuations, building pre-industrial to present-day timelines through phloem transport of foliage-derived Hg into annual xylem increments. However, species-specific anatomical factors can influence the extent to which tree rings accurately reflect historical atmospheric Hg exposure.

Advances in analytical methods have enhanced our ability to determine Hg sources and clarify the accumulation pathways in tree tissues. Stable isotope analyses now enable finer discrimination between various emission signatures (industrial, natural, and anthropogenic) and more detailed tracing of Hg transformations. Additionally, integrating Hg measurements with physiological indicators has improved our understanding of how basic plant processes (photosynthesis, transpiration, and nutrient uptake) interact with Hg acquisition, allowing for the exploration of potential Hg impacts at the cellular and ecosystem scales [22,23].

This review synthesises the recent progress in tree-based Hg biomonitoring across contemporary and historical timescales, emphasising the physiological, anatomical, and environmental controls that determine the accuracy of monitoring. We explored (i) the mechanistic factors influencing the exchange of Hg between the atmosphere and vegetation, (ii) the primary physiological factors determining foliar uptake and translocation in trees, (iii) methodologies for analysing Hg in leaves and tree rings, and (iv) advancements in analytical techniques that enhance interpretative capabilities. Furthermore, this study delineates the criteria necessary for effective Hg biomonitoring in both forest ecosystems and trees within urban and anthropized environments and suggests research priorities that align with the global objectives of the Minamata Convention.

## 2. Vegetation-Atmosphere Mercury Exchange: Biogeochemical and Ecological Significance

### 2.1. Atmospheric Mercury–Vegetation Exchange: Processes, Dynamics, and Cycling

Within forest ecosystems, soils dominate total Hg storage, accounting for 94–98% of the overall ecosystem content [24]. Although bark and other aboveground tissues may show relatively high Hg concentrations, the substantially greater mass of mineral soil, combined with litterfall-mediated Hg inputs, makes soil the ultimate repository for atmospheric Hg. Consequently, vegetation serves as a transient reservoir, channelling Hg from the atmosphere into terrestrial pools via foliage and litterfall.

**Figure 1:**
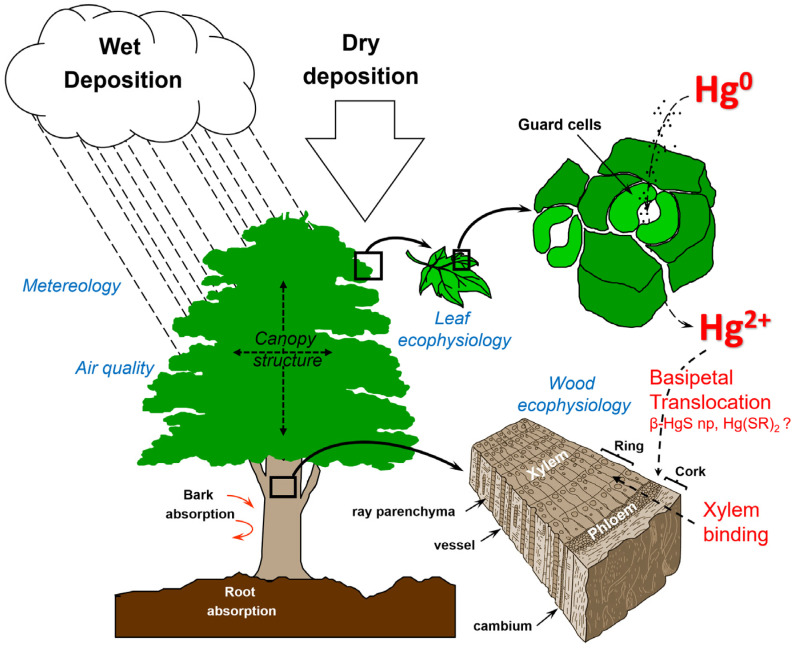
Conceptual model of Hg cycling in trees. Atmospheric Hg enters via wet and dry deposition, with Hg^0^ primarily taken up through leaf stomata, where it is oxidised to Hg^2+^. Canopy structure, meteorology, and air quality influence the deposition efficiency. Following uptake, Hg translocates basipetally (possibly as β-HgS nanoparticles and Hg(SR)_2_ complexes) and binds within woody tissues, essentially xylem. Sequential Hg incorporation into annual rings enables the dendrochronological reconstruction of historical atmospheric Hg levels.

The pivotal role of vegetation in global Hg cycling extends beyond its transient storage function. Forest canopies act as principal regulators of atmospheric Hg sequestration and are responsible for 60–90% of the total terrestrial deposition [5,7]. Annual foliar assimilation of atmospheric Hg (1010 ± 720 Mg yr^−1^) substantially outpaces wet deposition (590 ± 120 Mg yr^−1^), demonstrating the magnitude of vegetation-driven fluxes [5,6]. GEM underlies most of this exchange, with foliar uptake proceeding through stomatal diffusion, substomatal processing, and intracellular oxidation to Hg^2+^ [14,15]. This mechanism underpins the interface between the atmospheric Hg and terrestrial Hg cycles (Figure 1).

Vegetation–atmosphere Hg exchange drives marked seasonal variations in the ambient GEM concentrations. Observational studies have reported 30–60% lower GEM levels during peak growing seasons, closely mirroring the CO_2_ assimilation patterns [5,25]. In mid- to high-latitude forests, deciduous stands often attain atmospheric Hg uptake rates of 15–20 μg m^−2^ yr^−1^, significantly exceeding wet deposition inputs (4–8 μg m^−2^ yr^−1^) at comparable sites [16,26].

After foliar uptake, Hg follows three main pathways:

- Litterfall transfer (60–90% of total terrestrial Hg deposition): retained foliar Hg, including β-HgS nanoparticles and Hg(SR)_2_ complexes, ultimately enters forest soils upon senescence at rates of 10–34 μg m^−2^ yr^−1^ across diverse biomes [16]. The estimated global litterfall deposition ranges from 1180 to 1410 Mg yr^−1^ [6].
- Photoreduction and re-emission (10–25%): Under high light and temperature, foliar Hg can undergo photoreduction to GEM and re-enter the atmosphere, exhibiting strong diurnal cycles with peak emissions at midday [27,28]. This process has an exponential temperature dependence and is significantly enhanced by UVB radiation.
- Phloem translocation (5–15%): A smaller fraction is transported via the phloem to woody tissues, forming dendrochemical records of atmospheric Hg [23,25]. Multi-decadal tree-ring analyses documented a 2–3-fold increase in Hg during peak industrial emissions (1950–1980), followed by a gradual decline corresponding to regulatory efforts [29].

### 2.2. Environmental Modulators of Mercury Assimilation in Vegetation

Environmental conditions strongly influence Hg assimilation by vegetation. Multifactor regression analyses have revealed that temperature, humidity, and precipitation account for up to 43–67% of the variation in foliar Hg uptake across different forest biomes [20,30]. Among these factors, vapour pressure deficit (VPD) exerts a particularly strong effect by altering stomatal conductance. Uptake is maximised at moderate VPD (0.8–1.2 kPa), whereas extremely low (<0.5 kPa) or high (>2.0 kPa) values suppress stomatal opening and diminish Hg assimilation [28,30]. This nonlinear VPD response creates intricate diel and seasonal patterns and has implications for future forest Hg cycling under changing climatic conditions [31].

Water availability also mediates Hg assimilation. Moderate water stress (pre-dawn leaf water potential of −0.8 to −1.2 MPa) typically reduces Hg uptake by 25–40%, whereas severe drought (<−1.5 MPa) can result in a 60–85% reduction [32]. Interestingly, mild drought conditions may enhance Hg uptake via compensatory physiological responses [28]. Precipitation directly affects foliar Hg via the wash-off of surface-bound particulates and indirectly by altering stomatal conductance and atmospheric speciation [5,33]. Rainfall enhances Hg deposition, with throughfall concentrations 1.5–2 times higher than those of open-field rainfall. Vegetation intercepts approximately 67% of atmospheric Hg^2+^, although the exact reduction in Hg due to rainfall remains uncertain [33].

Additionally, atmospheric pollutants can modulate Hg assimilation by vegetation through various pathways. Elevated PM_2.5_ provides surfaces for Hg adsorption, altering leaf surface chemistry and potentially increasing stomatal uptake [21,34,35]. Reactive gases, such as NO_2_ and O_3_, may induce or inhibit stomatal opening and, in some cases, trigger oxidative stress, thereby modifying net Hg exchange [21,36,37]. Some pollutants also promote the in situ oxidation of Hg^0^ into more reactive forms, thereby enhancing foliar loading [21]. Consequently, these pollutant-driven effects can influence vegetation–Hg cycling, as they vary by species and functional type, reflecting differences in leaf morphology and physiology. 

### 2.3. Landscape- and Ecosystem-Scale Mercury Dynamics

Beyond stand-level factors, large-scale geographical and ecological contexts shape vegetation–Hg interactions. Altitudinal gradients can influence atmospheric GEM concentrations via enhanced mixing and long-range transport [38], whereas latitudinal shifts in photoperiod and growing season length produce distinct patterns of seasonal Hg uptake [25]. Topography further modulates local microclimates, leading to variability in foliar Hg accumulation along ridges and valleys that diverge from broader regional gradients [7].

At the biome scale, boreal forests act as major atmospheric Hg sinks, storing large amounts of Hg in organic soil horizons that are subject to microbial methylation [39,40]. Tropical forests, with high leaf area indices, are particularly effective at intercepting atmospheric Hg, especially near artisanal gold mining regions [41]. Temperate and montane forests occupy an intermediate position, with vertical stratification contributing to diverse Hg accumulation and transformation pathways [42,43].

Incorporating vegetation–atmosphere exchange into global Hg models has significantly enhanced predictive accuracy, reducing uncertainties by 35–60% compared to earlier models that focused on wet deposition [6]. Simulations suggest that without vegetation, the atmospheric residence time of GEM would increase from approximately 8 to 10 months, elevating annual Hg deposition to oceanic basins by nearly 960 Mg [6]. Vegetation thus emerges as a fundamental moderator of global Hg cycling, mitigating hemispheric gradients in atmospheric Hg and restricting inputs to marine environments [6,44]. This understanding of geographic, topographic, and ecosystem-level influences is crucial for designing biomonitoring strategies that accurately capture regional Hg trends and formulate robust management policies under a changing climate [42,45,46].

## 3. Mercury Ecophysiology in Trees: Uptake Mechanisms and Transport Pathways

### 3.1. Stomatal and Non-Stomatal Uptake Pathways

Hg is incorporated into trees through the foliar uptake of GEM for over 90% of Hg in aboveground biomass [25,47]. Multiple lines of evidence confirm this atmospheric pathway: stable isotope analyses consistently show mass-dependent fractionation (δ^202^Hg depleted by 2.1–2.8‰) in foliar tissues [25], whereas controlled exposure experiments reveal that soil-derived Hg contributes only 0.5–3.8% of aboveground Hg, even under artificially elevated soil levels [47]. Further validation comes from direct flux measurements documenting foliar GEM uptake rates of 0.05–3.7 ng m^−2^ h^−1^ across various ecosystems and seasons [6,20].

Stomatal absorption governs most GEM influx into leaves and is modulated by physiological factors such as stomatal conductance, leaf structure, and photosynthetic capacity (Figure 1) [28,31]. Deciduous broadleaves display higher instantaneous uptake rates but accumulate Hg over a single growing season, whereas evergreen conifers feature lower rates but retain their foliage for longer periods, facilitating multi-year Hg accumulation [21,48]. Non-stomatal (cuticular) diffusion remains minor under ambient GEM concentrations (<10% of total foliar Hg) because of the physicochemical properties of GEM, which favour stomatal over cuticular entry [48,49].

Other uptake routes (e.g., root or bark) generally contribute little to the aboveground Hg. Soil-derived Hg is largely immobilised in root systems owing to its complexation with soil organic matter and thiol-rich proteins [50,51]. However, bark can accumulate vapour-phase Hg (up to 77% of total bark Hg), with distinct biogenic processes such as nanoparticulate β-HgS formation occurring in the outer bark layers [52,53]. These findings confirm that bark may serve as an independent biomonitoring medium, although it reflects different accumulation mechanisms than foliage.

### 3.2. Leaf Morphological and Anatomical Determinants of Mercury Assimilation

Foliar structural and morphological attributes strongly influence GEM uptake, thereby shaping the efficiency with which vegetation captures atmospheric Hg. The specific leaf area (SLA) is positively correlated (r > 0.5, *p* < 0.01) with Hg accumulation rates in numerous tree species, suggesting that a larger surface area relative to volume promotes greater diffusion of Hg [14,21]. Mesophyll conductance also showed a positive correlation (r^2^ = 0.47, *p* < 0.01), emphasising the role of post-stomatal pathways in regulating Hg transport to oxidation and fixation sites within leaf tissues [20].

Assessments of foliar structural traits in 23 European tree species have identified negative correlations between Hg uptake efficiency and both leaf thickness (R^2^ = 0.39, *p* < 0.01) and cuticle thickness (R^2^ = 0.32, *p* < 0.05), suggesting that thicker leaves exert a higher diffusional resistance to GEM accumulation [20]. High-resolution imaging techniques have identified preferential Hg deposition around cuticular discontinuities and specialised epidermal structures, including stomatal guard cells [47,49].

Several experiments have shown strong positive correlations between stomatal aperture and Hg assimilation rates across different forest biomes, with photosynthetically active radiation (PAR)-mediated foliar Hg resistance reaching minima under optimal conditions (20 °C, 170 W m^−2^ irradiance) [54,55,56]. Diurnal studies using dynamic flux chambers have revealed that peak Hg uptake (ng m^−2^ h^−1^) often occurs in mid-morning (09:00–11:00), coinciding with maximum stomatal conductance (mol m^−2^ s^−1^). As the afternoon vapour pressure deficit increases, stomatal closure reduces Hg assimilation [57,58].

The tight linkage between C assimilation and Hg sequestration is supported by the observed correlations between seasonal CO_2_ uptake and GEM assimilation in temperate and boreal forest species [5,20]. These findings highlight the physiological integration of Hg uptake within broader plant–C relations, revealing a mechanistic basis for interpreting foliar Hg accumulation patterns.

### 3.3. Physiological and Functional Determinants of Species-Specific Mercury Accumulation

The functional type of vegetation critically influences the efficiency with which different species accumulate foliar Hg. Deciduous broadleaf species consistently exhibit higher Hg uptake than evergreen conifers, with mean accumulation rates in temperate forests reaching 0.21 ± 0.21 ng g^−1^ day^−1^ for broadleaves versus 0.10 ± 0.20 ng g^−1^ day^−1^ for conifers under similar atmospheric conditions [21]. At the ecosystem level, broadleaf forests show higher GEM deposition (25.1 µg m^−2^) than coniferous forests (13.4 µg m^−2^), reflecting substantial physiological differences, including a larger specific leaf area and higher stomatal conductance [20,21].

Although conifers have lower daily assimilation rates, their perennial leaf habit allows for extended Hg accumulation over the annual cycle, resulting in significant and long-term Hg uptake [23,59]. Tissue-specific patterns vary by species; in most cases, Hg concentrations are highest in mature foliage than in bark and woody tissues, although some species exhibit higher Hg levels in the bark than in the foliage [24,52]. Intraspecific variability often stems from microenvironmental gradients; for example, sun-exposed foliage within the same canopy can exhibit Hg levels that are 40–65% higher than those of shaded leaves [20,60]. Thus, standardised sampling protocols accounting for canopy position are essential for accurate foliar Hg biomonitoring.

Species-specific uptake capacities, coupled with seasonal changes in leaf physiology, produce characteristic temporal trajectories of foliar Hg. Peak concentrations typically occur just before leaf senescence, reflecting cumulative exposure over the growing season. The time-series data indicated three distinct accumulation phases:

1. Rapid initial uptake during leaf expansion (0.28–0.45 ng g^−1^ day^−1^)
2. Moderate accumulation under peak photosynthetic activity (0.12–0.26 ng g^−1^ day^−1^)
3. Reduced assimilation approaching senescence (0.03–0.09 ng g^−1^ day^−1^) [32,60].

As a result, late-season foliar Hg concentrations (25–58 ng g^−1^) can be four to eight times higher than those in newly emerged leaves (6–12 ng g^−1^) [14,20]. The overall monotonic increase supports the use of late-season foliage as an integrated metric for growing-season Hg exposure. Seasonal accumulation aligns with leaf phenology and photosynthetic performance, both of which drive the mechanistic coupling between carbon assimilation processes and Hg uptake [20,32].

### 3.4. Mercury Transport and Retention in Woody Tissues

Once formed in the foliage, Hg compounds are likely bound to thiol-containing organic molecules (Hg(SR)_2_) and transported to woody tissues largely via the phloem [25,61]. This process facilitates the downward movement of Hg into the developing xylem, where it can be retained in the tree rings [23]. Upon reaching the cambial zone, these complexes disperse radially into developing xylem cells, potentially via the ray parenchyma. Mercury is predominantly stored in cell walls, contributing to its long-term retention in tree tissues (Figure 1) [23,25,62]. Lignin-rich domains in the cell wall provide high-affinity binding sites for Hg(II), as demonstrated through EXAFS spectroscopy and surface complexation models [63]. Moreover, stress-induced lignin accumulation can enhance Hg immobilisation in vascular tissues, reinforcing the functional role of lignin in both detoxification and structural sequestration [64].

Mercury transport and retention vary significantly among different tree species. Gustin et al. [61] documented greater Hg variability in broadleaved angiosperms (*Populus*, *Quercus*) than gymnosperms (*Pinus* spp.), reflecting variations in vascular architecture, specifically xylem-phloem connectivity, and species-specific transpiration rates. McLagan et al. [25] and Peng et al. [23] observed distinct Hg retention patterns across conifer genera (*Pinus*, *Picea*, and *Larix*) correlating with differences in phloem loading efficiency and sapwood-heartwood transition dynamics [65].

Species-specific anatomical characteristics, particularly heartwood formation, lignification, and ray parenchyma structure, govern long-term Hg sequestration and intrawood translocation [23]. Although diffusion contributes to Hg movement, advective processes (typically 0.8–1.2 mm yr^−1^) often predominate in trees with extensive sapwood zones [66]. Consequently, Hg is not strictly confined to the growth ring corresponding to its year of deposition but can migrate radially into older rings through combined advection–diffusion mechanisms [66,67].

This radial mobility is especially pronounced in species with prolonged sapwood functionality and gradual heartwood transitions, such as *Pinus sylvestris*, where systematic shifts in Hg concentration can result in a chronological misalignment of deposition signals (as discussed in Section 5.2) [48,65]. In contrast, species that form heartwood more rapidly, such as *Larix decidua* and certain *Picea* spp., limit radial mobility and preserve more reliable annual Hg signals [25,68].

Tree age also modulates Hg dynamics in woody tissues. Rapidly growing juvenile trees may dilute recent Hg deposition in their outer rings due to vigorous xylem production [69], whereas mature trees, with declining metabolic activity in older tissues, exhibit altered xylem permeability, which influences Hg redistribution [66,70].

### 3.5. Climatic and Hydrological Influences on Mercury Partitioning in Trees

Climatic factors, particularly temperature and precipitation, play a crucial role in modulating both Hg uptake (see Section 2.2) and intra-plant translocation, significantly influencing tree-ring Hg concentrations [67]. For example, water stress may alter Hg transport by affecting phloem mobility and the efficiency of radial translocation [67,71,72]. Temperature fluctuations further impact Hg dynamics by modifying GEM oxidation rates and phloem viscosity, thereby influencing Hg mobility. Seasonal shifts in cambial activity contribute to interannual variability, independent of atmospheric Hg concentrations [20,66].

### 3.6. Integrated Effects of Physiological, Morphological, and Environmental Drivers

Multiple empirical models incorporate physiological and environmental predictors, such as stomatal conductance, photosynthetic rate, vapour pressure deficit, and atmospheric Hg levels, to explain species-specific foliar Hg accumulation [20,60]. These models achieved predictive accuracies of R^2^ = 0.62–0.86 (*p* < 0.001), highlighting the interlinked roles of morphological traits, taxonomic affiliation, and environmental conditions.

For instance, Zhang et al. [21] proposed a generalised model for coniferous species as follows:

THg_rate_ = 0.27 GEM − 0.53 Ws − 0.36 VWC + 0.40 PM_2.5_

where THg_rate_ is the foliar Hg accumulation rate, GEM is the atmospheric gaseous Hg concentration, Ws is the wind speed, VWC is the volumetric soil water content, and PM_2.5_ is fine particulate matter. Such formulations help integrate vegetation-driven Hg fluxes into broader biogeochemical cycles, aiding the prediction of ecosystem-level Hg dynamics [5,8].

Vertically resolved measurements further highlight the influence of canopy structure on atmospheric Hg gradients. Quant et al. [73] and Roy et al. [74] observed higher GEM concentrations above the canopy, with decreases within and below it, particularly during the daytime when foliar uptake intensifies. Nocturnal stratification can momentarily increase near-ground GEM concentrations owing to reduced mixing, underscoring the importance of both spatial and temporal dimensions in foliar Hg accumulation. Overall, these insights confirm that morphological, taxonomic, and temporal factors converge to shape foliar Hg dynamics, driving measurable intra- and interspecific variability and enriching our understanding of Hg cycling.

## 4. Tree Leaves as Biomonitors of Atmospheric Mercury

### 4.1. Historical Development and Conceptual Framework

By the early 1990s, scientists had recognised the potential of tree leaves to track atmospheric Hg. Initial research established the accumulation kinetics of foliar Hg under controlled conditions, followed by field studies that standardised sampling protocols and validated strong correlations between leaf Hg content and measured atmospheric levels [46,75,76]. Over time, improved analytical methods, such as thermal decomposition, have further increased the sensitivity and accuracy of leaf-based Hg biomonitoring. Validation studies comparing foliar Hg concentrations with conventional air monitoring have consistently confirmed significant correlations [77,78]. Collectively, these methodological refinements position tree leaves as valid and efficient biomonitors, offering insights into both temporal and spatial trends of atmospheric Hg.

### 4.2. Spatial-Temporal Assessment Using Leaf Sampling

Building on the foundational research and methodological refinements outlined in the previous section, leaf sampling has evolved into a robust approach for spatiotemporal assessments of atmospheric Hg. Tree leaves function as effective time-integrated biomonitors of Hg deposition across multiple spatiotemporal scales. The progressive accumulation of Hg in foliage throughout the growing season enables the robust characterisation of both short-term fluctuations and long-term trends in vegetation-atmosphere Hg exchange dynamics.

Foliar Hg concentrations exhibit characteristic temporal dynamics during the growing season, with accumulation rates that are closely linked to leaf development. Poissant et al. [79] observed that in a maple forest ecosystem, Hg concentrations in leaves increased from 8.7 ± 1.5 ng g^−1^ early in the season to 30.8 ± 3.0 ng g^−1^ by the end of the season, yielding a net accumulation of approximately 22.1 ng g^−1^ over approximately 135 days. This progressive enrichment was almost perfectly proportional to leaf age (R^2^ = 0.99, *p* < 0.001), underscoring the dominant role of foliar uptake in tree Hg assimilation. Although the study did not delineate distinct seasonal phases with variable daily accumulation rates or report correlations with photosynthetically active radiation and precipitation frequency, the continuous increase in foliar Hg concentrations demonstrates that maple forest canopies serve as effective sinks for atmospheric Hg accumulation.

Interannual monitoring at fixed sampling locations provides valuable insights into the long-term trends in atmospheric Hg deposition, which is a key metric for assessing regulatory effectiveness. McClenahen et al. [80] analysed archived and contemporary northern red oak (*Quercus rubra*) leaf samples from Pennsylvania and reported a robust linear decline in foliar Hg concentrations over the period from the mid-1980s to the early 2000s (R^2^ ≈ 0.81, *p* < 0.001). Their findings indicate that, particularly at sites with higher Hg exposure, late-season leaf Hg levels decreased by roughly 40–45%, a reduction that closely coincides with the implementation of the 1990 Clean Air Act amendments. This significant downward trend highlights the effectiveness of emissions controls and underscores the value of standardized foliar monitoring protocols for policy-relevant, temporal analyses of atmospheric Hg deposition

The time-integrated nature of foliar Hg accumulation enables the high-resolution spatial characterisation of atmospheric Hg deposition across heterogeneous landscapes. Regional-scale analyses across European forests have revealed pronounced spatial heterogeneity, with foliar Hg concentrations ranging from 12.3 to 42.7 ng g^−1^ at 232 sites [20]. Moreover, these spatial patterns correlated strongly with the modelled atmospheric concentrations of GEM (r = 0.76, *p* < 0.001), thereby validating foliar Hg as an effective proxy for atmospheric exposure.

Distance-decay functions characterise foliar Hg concentrations near anthropogenic emission sources. Gerson et al. [41] report a clear decrease in foliar Hg levels with increasing distance from active artisanal gold mining operations in the Peruvian Amazon. Similar spatial gradients have been observed in post-mining environments, where foliar sampling effectively characterizes airborne Hg contamination in urban settlements [81].

Leaf monitoring networks represent a cost-effective methodology for the high-resolution spatial assessment of atmospheric Hg deposition, complementing conventional instrumental monitoring approaches, particularly in regions with limited monitoring infrastructure.

### 4.3. Applications in Environmental Monitoring

By revealing key exposure pathways in populated regions, foliar Hg measurements provide essential data for ecological and human health risk assessments. Comparing washed versus unwashed foliage distinguishes between internally incorporated GEM and surface-bound particulate mercury (PBM), revealing atmospheric deposition processes and potential human transfer mechanisms [81]. Strong correlations between foliar Hg and human biomarkers have been reported in communities near artisanal gold mining or chlor-alkali industries, indicating that leaf-based measurements can serve as effective early warning indicators [41,81]. In policy contexts, decreases in foliar Hg concentrations following emission control measures provide empirical evidence of regulatory success [80,82]. Hence, leaf-based biomonitoring offers a low-cost, high-coverage tool for environmental management.

Recent methodological advances have illustrated the benefits of using conifer needles sampled over multiple years to reconstruct historical trends of Hg deposition. Franzaring et al. [83] documented consistent incremental increases in Hg concentrations across seven consecutive needle age classes (2016–2022), revealing the cumulative Hg incorporation patterns. This temporally resolved sampling approach transcends the limitations of single-year foliar assessments, providing retrospective insights that complement instrumental monitoring networks and enhance our mechanistic understanding of Hg accumulation trajectories in forest ecosystems.

In addition to these direct applications, integrating tree-based biomonitoring with existing instrumental networks can substantially broaden monitoring coverage, particularly in remote or resource-limited regions. For example, Wohlgemuth et al. [28] applied a bottom-up approach at 10 European research sites to quantify foliar Hg uptake fluxes by combining detailed foliar sampling with passive air sampler measurements of atmospheric GEM. Their findings demonstrated that forest foliage consistently absorbs GEM over the growing season, with uptake rates varying according to canopy height and needle age, and that foliar deposition exceeds wet deposition by a factor of four on average.

While passive air samplers are ideally suited for such integrative studies because of their ability to provide time-weighted average concentrations at low cost with good precision and high spatial resolution, their full potential has only recently begun to be explored. Novakova et al. [84] successfully used tree-ring archives to reconstruct historical atmospheric mercury levels, demonstrating the value of these complementary approaches. Additionally, Quant et al. [73] and Roy et al. [74] showed that passive samplers effectively detect seasonal GEM concentration patterns and vertical gradients within and above forest canopies, highlighting their utility in exploring canopy-related controls on GEM dynamics. However, both studies noted limitations in quantifying exchange fluxes owing to the influence of diurnal atmospheric conditions on time-integrated measurements.

This combined approach underscores the complementary utility of combining biomonitoring with both instrumental and passive sampling networks to achieve a more comprehensive understanding of Hg cycling in forested ecosystems, particularly in areas where conventional monitoring approaches are challenging to implement.

### 4.4. Methodological Considerations for Mercury Biomonitoring with Tree Leaves

Effective foliar biomonitoring relies on standardised protocols for sampling, processing, and analysis to allow cross-study comparisons. In deciduous species, late-season sampling (1–2 weeks before senescence) effectively captures cumulative Hg uptake, whereas in evergreen species, consistent sampling of a defined needle age class is essential to account for age-dependent variations in Hg accumulation [28,85,86]. Canopy position and solar aspect also influence foliar Hg concentrations because of the vertical and horizontal gradients in atmospheric Hg [73,74]. Outer crown leaves typically accumulate more Hg per unit area, reflecting higher stomatal conductance and greater physiological activity [14]. Standardising the sampling height and crown position reduces variability [20].

The selection of appropriate species for biomonitoring should consider both the monitoring objectives and the characteristics of the local flora. For instance, Zhang et al. [19] demonstrated that deciduous broadleaf species exhibit higher Hg concentrations on a mass basis, whereas evergreen needle species, despite their lower mass-based Hg levels, display significantly higher leaf mass per area (LMA) values. Consequently, when Hg accumulation is expressed per unit leaf area, evergreen conifers may accumulate comparable or even greater amounts of Hg than deciduous species [21]. Moreover, the use of conifers facilitates winter sampling, which is particularly valuable in regions that experience prolonged dry or warm periods [31]. For long-term or large-scale studies, selecting native species with well-characterised accumulation patterns will ensure consistent spatial comparisons. Collectively, these methodological considerations underpin robust foliar Hg biomonitoring in diverse ecosystems.

## 5. Tree Rings as Historical Archives of Atmospheric Mercury

### 5.1. Theoretical Framework for Dendrochronological Mercury Archives

Tree rings serve as annually resolved records of environmental change [87], preserving atmospheric Hg signals acquired during wood formation [88]. Although dendrochemical analyses of trace elements emerged in the 1970s [89], Hg-specific studies began more recently, following major analytical advances. Early studies in the 1990s, most notably Zhang et al. [90] on black spruce (*Picea mariana*) in boreal Quebec, first demonstrated that atmospheric inputs were the principal source of tree-ring Hg concentration. Following a period of limited research, the past decade has witnessed renewed interest in harnessing tree rings to reconstruct historical Hg deposition.

Compared to other environmental archives, such as ice cores, peat bogs, or lake sediments, tree rings predominantly capture GEM that enters the foliage through stomatal diffusion and is subsequently oxidised and translocated into the xylem (see Section 3.1 and Section 3.2) [66,91]. Their annual (or sub-annual) dating precision allows for the detection of localised pollution episodes and complements other high-resolution archives [84,92,93]. However, physiological processes, such as species-specific stomatal behaviour and phloem transport, can complicate data interpretation [94,95], making the choice of tree species and analytical methods critical for reliable chronological reconstructions.

**Figure 2:**
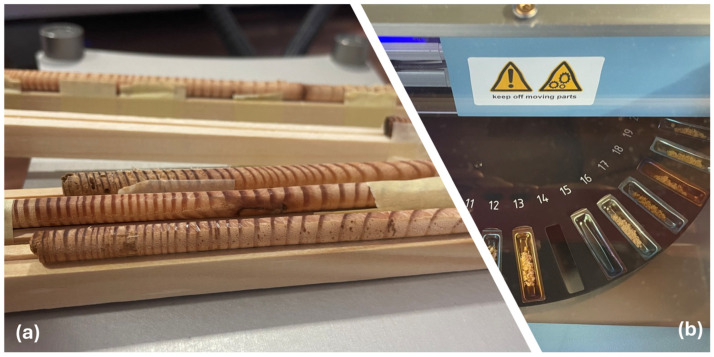
Methodology for dendrochronological Hg analysis. (**a**) Tree cores prepared for annual ring dating and measurement using a precision measuring stage. (**b**) Thermal Decomposition Amalgamation Atomic Absorption Spectroscopy (TDA-AAS) system (Milestone DMA-80^®^ Tri-Cells) for direct quantification of Hg in wood samples.

Recent methodological advances, including stable isotope analysis, refined understanding of Hg uptake and movement, and mathematical models accounting for radial translocation, have significantly improved the accuracy of tree-ring Hg chronologies (Figure 2) [66,96]. Such improvements highlight the importance of proper species selection and standardised analytical approaches for maximising the potential of Hg dendrochemistry to yield valuable historical perspectives on atmospheric Hg levels that complement and extend the evidence from other environmental archives.

### 5.2. Choosing Suitable Species and Minimizing Radial Translocation of Mercury

Species-specific anatomical features and growth patterns critically influence the fidelity of tree-ring Hg archives in dendrochronology. Conifers with narrow sapwood, particularly *Larix decidua* and certain *Picea* species, tend to display minimal radial redistribution as they rapidly form heartwood and limit metabolic activity in older rings [25,68]. In contrast, pines (*Pinus* spp.) often retain functional sapwood over many annual increments, promoting extensive radial Hg transport via ray parenchyma cells, which can displace concentration maxima away from their original calendar year [48,65]. In addition, high growth rates can dilute ring Hg content by expanding xylem volume, thereby masking atmospheric signals, whereas slower-growing specimens typically exhibit more distinct Hg gradients [23].

Radial translocation, primarily mediated by the ray parenchyma, can undermine the chronological integrity of dendrochemical reconstructions by shifting peak Hg concentrations by years or decades [66,97]. Once cells transition from sapwood to heartwood, their metabolic activity declines sharply, thereby reducing Hg mobility [25]. Consequently, monitoring the nitrogen content in wood may help flag rings subject to greater post-depositional movement because heightened metabolic processes generally correlate with increased translocation [23].

Physiological age further complicates dendrochemical records. In vigorously growing juvenile trees, rapid xylem production can skew outer rings toward higher apparent Hg levels if significant ongoing uptake and redistribution occur [25,97]. As trees mature and the heartwood fraction expands, the radial transport pathways gradually diminish [66,70]. Selecting species with rapid and consistent heartwood formation, such as *Larix decidua* or *Picea abies*, can substantially mitigate these translocation effects and maintain a more accurate temporal resolution [23,25,98].

While some studies indicate negligible Hg mobility in carefully chosen taxa [68], accumulating evidence confirms significant radial redistribution in certain *Pinus* species [66] and tropical gymnosperms with high annual increment rates [47]. Controlled experiments on *Pinus massoniana*, for example, have demonstrated that pronounced translocation in juvenile xylem can chronologically displace peak Hg signals relative to known emission timelines [67]. Together, these findings underscore the necessity of species-specific evaluations, addressing sapwood volume, heartwood formation patterns, and physiological traits to optimise tree-ring Hg archives for palaeoclimate reconstructions. Engaging such criteria facilitates robust long-term reconstructions of historical Hg deposition and strengthens our capacity to distinguish anthropogenic trends from natural variability [38,62].

### 5.3. Applications in Historical Reconstruction

**Figure 3:**
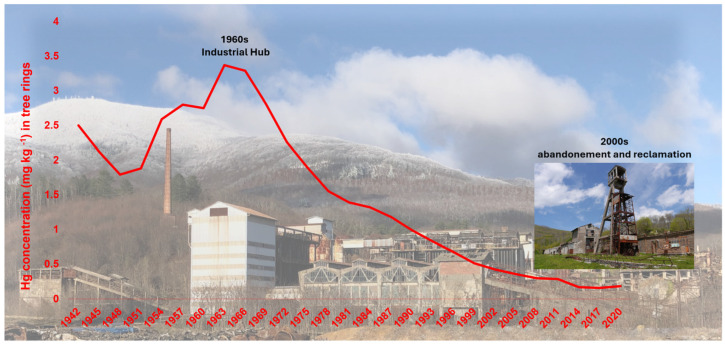
Dendrochronological reconstruction of atmospheric Hg contamination near the Abbadia San Salvatore mining district (Mt. Amiata, Italy). The red line quantifies Hg concentrations in *Tilia cordata* tree rings (mg kg^−1^), revealing remarkably high values (~3.5 mg kg^−1^) during peak industrial activity (1960s), followed by a progressive decline after mining operations ceased (data from [101]).

Dendrochronological Hg archives provide critical insights into anthropogenic impacts and guide environmental management strategies. [84]. For instance, Ghotra et al. [99] reported a 37% decline in tree-ring Hg concentrations in Canada’s Mackenzie Delta following the enactment of stricter emission controls, and McLagan et al. [25] observed parallel decreases in Central Europe concomitant with diminishing industrial activity. High-resolution records often reveal specific contamination episodes related to mining and metallurgy-related activities. For example, in the Klondike gold fields, pronounced Hg peaks correspond to periods of intense amalgamation-based extraction [100], whereas in Central Europe, temporal maxima in tree-ring Hg align with historical peaks in industrial output [84]. In Italy, Baroni et al. [101] documented abrupt Hg reductions following mine closures in a legacy mining district (Figure 3), and Fornasaro et al. [102] employed *Castanea sativa* dendrochronologies to reconstruct extended pollution histories in the Monte Amiata region. Their findings underscore the strong archival capacity of this broadleaf species to track long-term Hg exposure and assess persistent environmental contamination.

Industrial point sources have also been effectively studied using dendrochemical analyses. Chlor-alkali plants, for instance, commonly generate distinctive Hg signatures in tree rings [103], whereas *Larix decidua* chronologies have reliably traced emissions from gold amalgamation, chemical processing, and metal refining [59,68]. In parallel, some studies have distinguished natural or geogenic contributions, such as volcanic events in the Canary Islands [104,105]. Moreover, regional-scale reconstructions in Tasmania [106] and Austria [98] validated dendrochronological dating by correlating elevated Hg signals with surges in smelting activity. These applications collectively underscore the value of tree-ring Hg data in revealing both localised and regional pollution histories across diverse temporal and spatial scales.

### 5.4. Analytical Methodologies for Tree-Ring Mercury Quantification

Dendrochemical studies rely on precise, high-resolution measurements to assess the annual Hg input recorded in tree rings. The fundamental principle is that each growth ring reflects a single growing season. Accurate ring boundary identification (cross-dating) and sampling of individual annual growth layers are the basis for a robust dendrochronological analysis [48]. Achieving this level of precision requires the following:

- Rigorous cross-dating procedures to confirm the exact calendar year of each ring.
- High-resolution sampling, preferably at annual or biennial scales, to capture fine temporal trends in atmospheric Hg uptake.
- Elimination of potential contamination through careful sample preparation and handling.

Thermal Decomposition Amalgamation Atomic Absorption Spectroscopy (TDA-AAS) remains one of the principal methods for quantifying Hg in tree rings, offering detection limits of approximately 1–5 ng g^−1^ while avoiding acid digestion [25]. Its direct measurement approach facilitates rapid sample throughput and is particularly advantageous when tree rings are clearly delineated, thereby minimising the required sample mass for analysis. In contrast, cold vapour atomic fluorescence spectrometry (CVAFS) provides higher sensitivity but requires more extensive sample preparation (e.g., acid digestion and chemical reduction), which can increase contamination risks if not carefully managed. Inductively coupled plasma mass spectrometry (ICP-MS) is widely used for broader dendrochemical analyses but generally demonstrates lower sensitivity and resolution relative to TDA-AAS or CVAFS. Although methods such as cysteine addition can mitigate sample loss, these strategies do not fully offset the analytical drawbacks of ICP-MS [107,108].

Laser ablation inductively coupled plasma mass spectrometry (LA-ICP-MS) offers high-resolution spatial mapping (10–50 µm spot size) of Hg within or across rings. By scanning radially, this technique can detect intra-ring heterogeneity and localised accumulations that bulk analyses might average out [109,110]. Such spatially resolved data can reveal short-term Hg events or subtle shifts in seasonal assimilation patterns; however, accurate ring boundary delineation remains a critical challenge.

Stable Hg isotope analyses (e.g., δ^202^Hg and Δ^199^Hg) are increasingly used to differentiate industrial sources from natural sources and to investigate physiological assimilation pathways. However, as Gačnik et al. [111] noted, the extent to which plant uptake processes, particularly through leaves and woody tissues, may alter atmospheric Hg isotopic signatures remains unclear. Their synthesis underscores both the promise and current limitations of using biological archives to uncover source-specific isotope patterns. In addition to strengthening source apportionment studies, isotopic signatures also help determine whether radial Hg mobility modifies the original signal ascribed to a particular growth year [25,69,96].

Hg isotopes and plant functional markers (e.g., δ^13^C and δ^18^O) may help establish mechanistic links between stomatal processes, water-use efficiency [22], and Hg uptake and incorporation into wood. For instance, analysing δ^13^C in parallel with Hg may clarify how changes in stomatal conductance (e.g., due to drought stress) influence the magnitude of atmospheric Hg assimilation.

Mathematical modelling approaches, such as advection-diffusion frameworks, are used to account for radial Hg translocation within stems and correct for chronological distortions [66,67]. Evidence of potential Hg movement across ring boundaries underscores the importance of comparing modelled “corrected” chronologies with raw ring-specific data, particularly for species known to exhibit enhanced radial mobility (e.g., certain pine species).

Finally, the N content in tree rings, an indirect metric of metabolically active tissues, has been suggested as a marker for identifying increments with a higher Hg translocation potential [23]. Integrating chemical (N, isotopes), anatomical (sapwood-heartwood boundary), and modelling approaches can significantly enhance the temporal accuracy and interpretive power of tree-ring Hg reconstruction.

In conclusion, reliable tree-ring Hg analyses depend on the following factors:

- Annual-resolution ring separation through rigorous cross-dating,
- Careful method selection (TDA-AAS, CVAFS, LA-ICP-MS, or stable isotope approaches) matched to the study’s resolution and sensitivity needs, and
- Physiological data integration to assess species-specific mobility effects.

**Figure 4:**
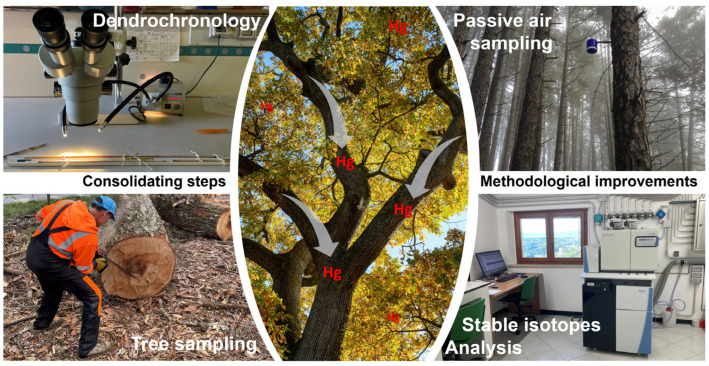
Schematic representation of the key methodological steps in tree-based biomonitoring of atmospheric Hg deposition. Passive air sampling and advancements in stable isotope analysis are highlighted as critical improvements for refining atmospheric mercury monitoring.

When combined, these strategies yield more accurate reconstructions of atmospheric Hg trends, thereby advancing our understanding of historical contamination events and the efficacy of emission-control measures (Figure 4).

## 6. Future Directions and Conclusions

### 6.1. Integrative Perspectives on Leaves and Tree Rings

Trees provide multitemporal chronologies of atmospheric Hg; foliage responds rapidly to fluctuations in GEM, whereas tree rings preserve historical deposition signatures. For example, both *Pinus nigra* and *Populus tremuloides* exhibit short-term foliar fluctuations of Hg within days or weeks [47], whereas dendrochemical records extend over decades and capture legacy contaminations [38]. The integration of these two approaches elucidates both contemporary variability and long-term trends in Hg cycling.

Interspecific differences significantly influence the dynamics of Hg bioaccumulation. Under equivalent exposure conditions, *Populus* foliage typically accumulates higher Hg concentrations than *Pinus* foliage, reflecting fundamental disparities in leaf morphology and stomatal conductance pathways [47]. Similarly, bark, leaves, and wood differentially sequester distinct Hg species, particularly in environments where GOM predominates [38]. Standardised coring and leaf sampling protocols are essential for ensuring cross-site comparability and analytical reliability [25,99].

### 6.2. Ecophysiological Integration

Physiological mechanisms, including stomatal conductance, phloem loading, and radial transport, fundamentally regulate Hg sequestration in foliar tissues and the xylem [20,23]. Even phylogenetically proximate taxa exhibit differential uptake patterns attributable to morphological variations [47], epicuticular wax composition, and cuticle thickness (Zhang et al., 2025) [21]. Additionally, atmospheric contaminants, such as PM_2.5_ and NO_2_, can enhance stomatal aperture or compromise cuticular integrity, consequently modulating Hg assimilation rates. The integration of ecophysiological proxies (e.g., δ^13^C, δ^18^O, and xylem sap flux) with pollution indices facilitates the differentiation between authentic atmospheric Hg trends and confounding environmental stressors, including chronic exposure to PM or NO_2_.

From a retrospective analytical framework, such integrated multiparametric datasets derived from foliar and dendrochronological archives facilitate mechanistic determination of whether observed Hg concentration gradients reflect physiological adaptations, atmospheric contaminant dynamics, or broader climatic variables, thereby enabling more robust attribution of temporal and spatial variability in dendrochemical and foliar Hg records.

### 6.3. Climate Change Implications

Elevated temperatures can enhance stomatal conductance, consequently amplifying the foliar Hg uptake rate [20,112]. However, drought conditions induced by warming may reduce stomatal conductance, thereby limiting gross ecosystem metabolism and GEM assimilation [56]. Concurrently, climate-driven shifts in vegetation communities toward drought-tolerant species can fundamentally alter leaf morphological and physiological traits, thereby modifying the overall Hg bioaccumulation capacities [60]. Permafrost degradation mobilises legacy Hg deposits, potentially confounding dendrochemical chronologies by elevating ambient baseline concentrations [20]. Extended multi-species field trials are vital for clarifying these processes under diverse climatic conditions [56,60].

### 6.4. Policy Relevance Under the Minamata Convention

Foliar and dendrochronological biomonitoring supports the implementation of the Minamata Convention by providing data from regions with limited measurement infrastructure. Foliage functions as a sensor for present atmospheric Hg concentrations, whereas tree rings provide historical documentation of deposition patterns across decades to centuries. Effective implementation requires harmonised sampling protocols and cross-laboratory validation aligned with international standards. These tree-based Hg records strengthen global assessments, inform evidence-based risk management, and guide the refinement of Hg reduction targets.

### 6.5. Conclusions

Dual biomonitoring, integrating current foliar measurements with dendrochemical reconstructions, provides comprehensive insights into Hg cycling within forest ecosystems and anthropized environments, where trees near emission sources provide valuable monitoring data. Methodological challenges persist, particularly regarding radial translocation effects and interspecific variability in the foliar assessment. However, advances in sampling techniques, analytical methods, and ecophysiological modelling are improving the temporal resolution and data reliability. As anthropogenic emission profiles evolve and climate change progresses, tree-derived chronologies remain essential for documenting atmospheric Hg trends, evaluating remediation effectiveness, and revealing critical vegetation-atmosphere Hg exchange processes.

## Data Availability

This study did not involve the generation of new data.

## Abbreviations

The following abbreviations are used in this manuscript:

GEM	Gaseous Elemental Mercury
GOM	Gaseous Oxidased Mercury
PBM	Particulate-Bound Mercury

## References

[1] Augusto L., Boča A. (2022). Tree Functional Traits, Forest Biomass, and Tree Species Diversity Interact with Site Properties to Drive Forest Soil Carbon. Nat. Commun..

[2] Ma H., Crowther T.W., Mo L., Maynard D.S., Renner S.S., Van Den Hoogen J., Zou Y., Liang J., de-Miguel S., Nabuurs G.-J. (2023). The Global Biogeography of Tree Leaf Form and Habit. Nat. Plants.

[3] Chen X., Taylor A.R., Reich P.B., Hisano M., Chen H.Y.H., Chang S.X. (2023). Tree Diversity Increases Decadal Forest Soil Carbon and Nitrogen Accrual. Nature.

[4] Dubbert M., Werner C. (2019). Water Fluxes Mediated by Vegetation: Emerging Isotopic Insights at the Soil and Atmosphere Interfaces. New Phytol..

[5] Jiskra M., Sonke J.E., Obrist D., Bieser J., Ebinghaus R., Myhre C.L., Pfaffhuber K.A., Wängberg I., Kyllönen K., Worthy D. (2018). A Vegetation Control on Seasonal Variations in Global Atmospheric Mercury Concentrations. Nat. Geosci..

[6] Zhou J., Obrist D., Dastoor A., Jiskra M., Ryjkov A. (2021). Vegetation Uptake of Mercury and Impacts on Global Cycling. Nat. Rev. Earth Environ..

[7] Wang X., Yuan W., Lin C.-J., Feng X. (2022). Mercury Cycling and Isotopic Fractionation in Global Forests. Crit. Rev. Environ. Sci. Technol..

[8] Wu Q., Zhang Y., Li P., Fu X., Zhang Q., Wang X., Chen L., Wang S., Wang F., Feng X. (2022). Ecosystem Mercury Recovery and Health Benefit Under the Minamata Convention in a Changing Climate. Rev. Environ. Contam..

[9] Zhang Y., Song Z., Huang S., Zhang P., Peng Y., Wu P., Gu J., Dutkiewicz S., Zhang H., Wu S. (2021). Global Health Effects of Future Atmospheric Mercury Emissions. Nat. Commun..

[10] Lyman S.N., Cheng I., Gratz L.E., Weiss-Penzias P., Zhang L. (2020). An Updated Review of Atmospheric Mercury. Sci. Total Environ..

[11] Sonke J.E., Angot H., Zhang Y., Poulain A., Björn E., Schartup A. (2023). Global Change Effects on Biogeochemical Mercury Cycling. Ambio.

[12] Horowitz H.M., Jacob D.J., Zhang Y., Dibble T.S., Slemr F., Amos H.M., Schmidt J.A., Corbitt E.S., Marais E.A., Sunderland E.M. (2017). A New Mechanism for Atmospheric Mercury Redox Chemistry: Implications for the Global Mercury Budget. Atmos. Chem. Phys..

[13] Obrist D., Kirk J.L., Zhang L., Sunderland E.M., Jiskra M., Selin N.E. (2018). A Review of Global Environmental Mercury Processes in Response to Human and Natural Perturbations: Changes of Emissions, Climate, and Land Use. Ambio.

[14] Laacouri A., Nater E.A., Kolka R.K. (2013). Distribution and Uptake Dynamics of Mercury in Leaves of Common Deciduous Tree Species in Minnesota, U.S.A. Environ. Sci. Technol..

[15] Manceau A., Wang J., Rovezzi M., Glatzel P., Feng X. (2018). Biogenesis of Mercury–Sulfur Nanoparticles in Plant Leaves from Atmospheric Gaseous Mercury. Environ. Sci. Technol..

[16] Wang X., Bao Z., Lin C.-J., Yuan W., Feng X. (2016). Assessment of Global Mercury Deposition through Litterfall. Environ. Sci. Technol..

[17] Zhou J., Bollen S.W., Roy E.M., Hollinger D.Y., Wang T., Lee J.T., Obrist D. (2023). Comparing Ecosystem Gaseous Elemental Mercury Fluxes over a Deciduous and Coniferous Forest. Nat. Commun..

[18] Feinberg A., Dlamini T., Jiskra M., Shah V., Selin N.E. (2022). Evaluating Atmospheric Mercury (Hg) Uptake by Vegetation in a Chemistry-Transport Model. Environ. Sci. Process. Impacts.

[19] Obrist D., Roy E.M., Harrison J.L., Kwong C.F., Munger J.W., Moosmueller H., Romero C.D., Sun S., Zhou J., Commane R. (2021). Previously Unaccounted Atmospheric Mercury Deposition in a Midlatitude Deciduous Forest. Proc. Natl. Acad. Sci. USA.

[20] Wohlgemuth L., Rautio P., Ahrends B., Russ A., Vesterdal L., Waldner P., Timmermann V., Eickenscheidt N., Fürst A., Greve M. (2022). Physiological and Climate Controls on Foliar Mercury Uptake by European Tree Species. Biogeosciences.

[21] Zhang X., Kang H., Liu X., Zhou J., Liu M., Wang L., Xing X., Lu Q., Zeng X., Wei N. (2025). Comparative Foliar Atmospheric Mercury Accumulation across Functional Types in Temperate Trees. Environ. Sci. Technol..

[22] Siegwolf R.T.W., Lehmann M.M., Goldsmith G.R., Churakova (Sidorova) O.V., Mirande-Ney C., Timoveeva G., Weigt R.B., Saurer M. (2023). Updating the Dual C and O Isotope—Gas-exchange Model: A Concept to Understand Plant Responses to the Environment and Its Implications for Tree Rings. Plant Cell Environ..

[23] Peng H., Zhang X., Bishop K., Marshall J., Nilsson M.B., Li C., Björn E., Zhu W. (2024). Tree Rings Mercury Controlled by Atmospheric Gaseous Elemental Mercury and Tree Physiology. Environ. Sci. Technol..

[24] Sa R., Wang Z., Xu Z., Zhao Q., Zhang Q., Zhang X. (2023). Distribution Characteristics of Mercury Concentration and Estimation of Mercury Pools in Different Age Groups of *Larix gmelinii* Forests of Daxing’an Mountain. Environ. Pollut..

[25] McLagan D.S., Biester H., Navrátil T., Kraemer S.M., Schwab L. (2022). Internal Tree Cycling and Atmospheric Archiving of Mercury: Examination with Concentration and Stable Isotope Analyses. Biogeosciences.

[26] Demers J.D., Blum J.D., Zak D.R. (2013). Mercury Isotopes in a Forested Ecosystem: Implications for Air-surface Exchange Dynamics and the Global Mercury Cycle. Glob. Biogeochem. Cycles.

[27] Yuan W., Sommar J., Lin C.-J., Wang X., Li K., Liu Y., Zhang H., Lu Z., Wu C., Feng X. (2019). Stable Isotope Evidence Shows Re-Emission of Elemental Mercury Vapor Occurring after Reductive Loss from Foliage. Environ. Sci. Technol..

[28] Wohlgemuth L., Osterwalder S., Joseph C., Kahmen A., Hoch G., Alewell C., Jiskra M. (2020). A Bottom-up Quantification of Foliar Mercury Uptake Fluxes across Europe. Biogeosciences.

[29] Liu X., Wang X., Yuan W., Wang D., Feng X. (2023). Tree Rings Recording Historical Atmospheric Mercury: A Review of Progresses and Challenges. Crit. Rev. Environ. Sci. Technol..

[30] Yuan T., Huang S., Zhang P., Song Z., Ge J., Miao X., Wang Y., Pang Q., Peng D., Wu P. (2024). Potential Decoupling of CO2 and Hg Uptake Process by Global Vegetation in the 21st Century. Nat. Commun..

[31] Wohlgemuth L., Feinberg A., Buras A., Jiskra M. (2023). A Spatial Assessment of Current and Future Foliar Hg Uptake Fluxes Across European Forests. Glob. Biogeochem. Cycles.

[32] Teixeira D.C., Lacerda L.D., Silva-Filho E.V. (2018). Foliar Mercury Content from Tropical Trees and Its Correlation with Physiological Parameters in Situ. Environ. Pollut..

[33] Yuan W., Wang X., Lin C.-J., Zhang G., Wu F., Liu N., Jia L., Zhang H., Lu H., Dong J. (2024). Fate and Transport of Mercury through Waterflows in a Tropical Rainforest. Environ. Sci. Technol..

[34] Chen D., Yin S., Zhang X., Lyu J., Zhang Y., Zhu Y., Yan J. (2022). A High-Resolution Study of PM2.5 Accumulation inside Leaves in Leaf Stomata Compared with Non-Stomatal Areas Using Three-Dimensional X-Ray Microscopy. Sci. Total Environ..

[35] Steinparzer M., Schaubmayr J., Godbold D.L., Rewald B. (2023). Particulate Matter Accumulation by Tree Foliage Is Driven by Leaf Habit Types, Urbanization- and Pollution Levels. Environ. Pollut..

[36] Xu S., Li B., Li P., He X., Chen W., Yan K., Li Y., Wang Y. (2019). Soil High Cd Exacerbates the Adverse Impact of Elevated O_3_ on *Populus alba* “*Berolinensis*” L.. Ecotoxicol. Environ. Saf..

[37] Goyal D., Yadav A., Vats T., Saxena P., Srivastava A. (2020). Air Pollution and Its Role in Stress Physiology. Air Pollution and Environmental Health.

[38] Peckham M.A., Gustin M.S., Weisberg P.J. (2019). Assessment of the Suitability of Tree Rings as Archives of Global and Regional Atmospheric Mercury Pollution. Environ. Sci. Technol..

[39] Jiskra M., Wiederhold J.G., Skyllberg U., Kronberg R.-M., Hajdas I., Kretzschmar R. (2015). Mercury Deposition and Re-Emission Pathways in Boreal Forest Soils Investigated with Hg Isotope Signatures. Environ. Sci. Technol..

[40] Landis J.D., Obrist D., Zhou J., Renshaw C.E., McDowell W.H., Nytch C.J., Palucis M.C., Del Vecchio J., Montano Lopez F., Taylor V.F. (2024). Quantifying Soil Accumulation of Atmospheric Mercury Using Fallout Radionuclide Chronometry. Nat. Commun..

[41] Gerson J.R., Szponar N., Zambrano A.A., Bergquist B., Broadbent E., Driscoll C.T., Erkenswick G., Evers D.C., Fernandez L.E., Hsu-Kim H. (2022). Amazon Forests Capture High Levels of Atmospheric Mercury Pollution from Artisanal Gold Mining. Nat. Commun..

[42] Zeng S., Wang X., Yuan W., Luo J., Wang D. (2022). Mercury Accumulation and Dynamics in Montane Forests along an Elevation Gradient in Southwest China. J. Environ. Sci..

[43] Gerson J.R., Driscoll C.T., Demers J.D., Sauer A.K., Blackwell B.D., Montesdeoca M.R., Shanley J.B., Ross D.S. (2017). Deposition of Mercury in Forests across a Montane Elevation Gradient: Elevational and Seasonal Patterns in Methylmercury Inputs and Production. JGR Biogeosciences.

[44] Wang X., Luo J., Yuan W., Lin C.-J., Wang F., Liu C., Wang G., Feng X. (2020). Global Warming Accelerates Uptake of Atmospheric Mercury in Regions Experiencing Glacier Retreat. Proc. Natl. Acad. Sci. USA.

[45] Woś B., Gruba P., Socha J., Pietrzykowski M. (2021). Biomonitoring of Mercury Contamination in Poland Based on Its Concentration in Scots Pine (*Pinus sylvestris* L.) Foliage. Int. J. Environ. Res. Public Health.

[46] Lodenius M. (2013). Use of Plants for Biomonitoring of Airborne Mercury in Contaminated Areas. Environ. Res..

[47] Arnold J., Gustin M.S., Weisberg P.J. (2018). Evidence for Nonstomatal Uptake of Hg by Aspen and Translocation of Hg from Foliage to Tree Rings in Austrian Pine. Environ. Sci. Technol..

[48] Gačnik J., Gustin M.S. (2023). Tree Rings as Historical Archives of Atmospheric Mercury: A Critical Review. Sci. Total Environ..

[49] Stamenkovic J., Gustin M.S. (2009). Nonstomatal versus Stomatal Uptake of Atmospheric Mercury. Environ. Sci. Technol..

[50] Bishop K.H., Lee Y.-H., Munthe J., Dambrine E. (1998). Xylem Sap as a Pathway for Total Mercury and Methylmercury Transport from Soils to Tree Canopy in the Boreal Forest. Biogeochmistry.

[51] Yanai R.D., Yang Y., Wild A.D., Smith K.T., Driscoll C.T. (2020). New Approaches to Understand Mercury in Trees: Radial and Longitudinal Patterns of Mercury in Tree Rings and Genetic Control of Mercury in Maple Sap. Water Air Soil. Pollut..

[52] Liu Y., Lin C.-J., Yuan W., Lu Z., Feng X. (2021). Translocation and Distribution of Mercury in Biomasses from Subtropical Forest Ecosystems: Evidence from Stable Mercury Isotopes. Acta Geochim..

[53] Bardelli F., Rimondi V., Lattanzi P., Rovezzi M., Isaure M.-P., Giaccherini A., Costagliola P. (2022). *Pinus nigra* Bark from a Mercury Mining District Studied with High Resolution XANES Spectroscopy. Environ. Sci. Process. Impacts.

[54] Rutter A.P., Schauer J.J., Shafer M.M., Creswell J., Olson M.R., Clary A., Robinson M., Parman A.M., Katzman T.L. (2011). Climate Sensitivity of Gaseous Elemental Mercury Dry Deposition to Plants: Impacts of Temperature, Light Intensity, and Plant Species. Environ. Sci. Technol..

[55] Naharro R., Maria Esbri J., Angel Amoros J., Higueras P.L. (2020). Experimental Assessment of the Daily Exchange of Atmospheric Mercury in *Epipremnum aureum*. Environ. Geochem. Health.

[56] Yang Y.H., Kim M.-S., Park J., Kwon S.Y. (2024). Atmospheric Mercury Uptake and Accumulation in Forests Dependent on Climatic Factors. Environ. Sci. Process. Impacts.

[57] Sommar J., Zhu W., Shang L., Lin C.-J., Feng X. (2016). Seasonal Variations in Metallic Mercury (Hg^0^) Vapor Exchange over Biannual Wheat–Corn Rotation Cropland in the North China Plain. Biogeosciences.

[58] Converse A.D., Riscassi A.L., Scanlon T.M. (2010). Seasonal Variability in Gaseous Mercury Fluxes Measured in a High-Elevation Meadow. Atmos. Environ..

[59] Navrátil T., Šimeček M., Shanley J.B., Rohovec J., Hojdová M., Houška J. (2017). The History of Mercury Pollution near the Spolana Chlor-Alkali Plant (Neratovice, Czech Republic) as Recorded by Scots Pine Tree Rings and Other Bioindicators. Sci. Total Environ..

[60] Sun M., Yuan W., Liu N., Jia L., Wu F., Huang J.-H., Wang X., Feng X. (2025). Combined Impacts of Climate and Tree Physiology on Mercury Accumulation in Tropical and Subtropical Foliage and Robust Model Parametrization. Environ. Sci. Technol..

[61] Gustin M.S., Ingle B., Dunham-Cheatham S.M. (2022). Further Investigations into the Use of Tree Rings as Archives of Atmospheric Mercury Concentrations. Biogeochemistry.

[62] Gustin M.S., Dunham-Cheatham S.M., Harper J.F., Choi W.-G., Blum J.D., Johnson M.W. (2022). Investigation of the Biochemical Controls on Mercury Uptake and Mobility in Trees. Sci. Total Environ..

[63] Lv J., Luo L., Zhang J., Christie P., Zhang S. (2012). Adsorption of Mercury on Lignin: Combined Surface Complexation Modeling and X-Ray Absorption Spectroscopy Studies. Environ. Pollut..

[64] Shao R., Zhang J., Shi W., Wang Y., Tang Y., Liu Z., Sun W., Wang H., Guo J., Meng Y. (2022). Mercury Stress Tolerance in Wheat and Maize Is Achieved by Lignin Accumulation Controlled by Nitric Oxide. Environ. Pollut..

[65] Nováková T., Navrátil T., Demers J.D., Roll M., Rohovec J. (2021). Contrasting Tree Ring Hg Records in Two Conifer Species: Multi-Site Evidence of Species-Specific Radial Translocation Effects in Scots Pine versus European Larch. Sci. Total Environ..

[66] Chellman N., Csank A., Gustin M.S., Arienzo M.M., Vargas Estrada M., McConnell J.R. (2020). Comparison of Co-Located Ice-Core and Tree-Ring Mercury Records Indicates Potential Radial Translocation of Mercury in Whitebark Pine. Sci. Total Environ..

[67] Liu X., Wang X., Wang D. (2024). Assessment of Tree-Ring Mercury Radial Translocation and Age Effect in Masson Pine: Implications for Historical Atmospheric Mercury Reconstruction. J. Environ. Sci..

[68] Navrátil T., Nováková T., Shanley J.B., Rohovec J., Matoušková Š., Vaňková M., Norton S.A. (2018). Larch Tree Rings as a Tool for Reconstructing 20th Century Central European Atmospheric Mercury Trends. Environ. Sci. Technol..

[69] Wang X., Yuan W., Lin C.-J., Wu F., Feng X. (2021). Stable Mercury Isotopes Stored in Masson Pinus Tree Rings as Atmospheric Mercury Archives. J. Hazard. Mater..

[70] Cutter B., Guyette R. (1993). Anatomical, Chemical, and Ecological Factors Affecting Tree Species Choice in Dendrochemistry Studies. J. Environ. Qual..

[71] Lehmann M.M., Schuler P., Cormier M.-A., Allen S.T., Leuenberger M., Voelker S., Siegwolf R.T.W., Brooks J.R., Roden J., Saurer M. (2022). The Stable Hydrogen Isotopic Signature: From Source Water to Tree Rings. Stable Isotopes in Tree Rings.

[72] Dannoura M., Epron D., Desalme D., Massonnet C., Tsuji S., Plain C., Priault P., Gérant D. (2019). The Impact of Prolonged Drought on Phloem Anatomy and Phloem Transport in Young Beech Trees. Tree Physiol..

[73] Quant M.I., Feigis M., Mistry S., Lei Y.D., Mitchell C.P.J., Staebler R., Di Guardo A., Terzaghi E., Wania F. (2021). Using Passive Air Samplers to Quantify Vertical Gaseous Elemental Mercury Concentration Gradients Within a Forest and Above Soil. JGR Atmos..

[74] Roy E.M., Zhou J., Wania F., Obrist D. (2023). Use of Atmospheric Concentrations and Passive Samplers to Assess Surface-Atmosphere Exchange of Gaseous Mercury in Forests. Chemosphere.

[75] Gaggi C., Chemello G., Bacci E. (1991). Mercury Vapour Accumulation in Azalea Leaves. Chemosphere.

[76] Loppi S., Nelli L., Ancora S., Bargagli R. (1997). Passive Monitoring of Trace Elements by Means of Tree Leaves, Epiphytic Lichens and Bark Substrate. Environ. Monit. Assess..

[77] Barquero J.I., Rojas S., Esbrí J.M., García-Noguero E.M., Higueras P. (2019). Factors Influencing Mercury Uptake by Leaves of Stone Pine (*Pinus pinea* L.) in Almadén (Central Spain). Environ. Sci. Pollut. Res..

[78] Fantozzi L., Ferrara R., Dini F., Tamburello L., Pirrone N., Sprovieri F. (2013). Study on the Reduction of Atmospheric Mercury Emissions from Mine Waste Enriched Soils through Native Grass Cover in the Mt. Amiata Region of Italy. Environ. Res..

[79] Poissant L., Pilote M., Yumvihoze E., Lean D. (2008). Mercury Concentrations and Foliage/Atmosphere Fluxes in a Maple Forest Ecosystem in Québec, Canada. J. Geophys. Res..

[80] McClenahen J.R., Hutnik R.J., Davis D.D. (2013). Spatial and Temporal Patterns of Bioindicator Mercury in Pennsylvania Oak Forest. J. Environ. Qual..

[81] Monaci F., Ancora S., Paoli L., Loppi S., Franzaring J. (2023). Air Quality in Post-Mining Towns: Tracking Potentially Toxic Elements Using Tree Leaves. Environ. Geochem. Health.

[82] Viso S., Rivera S., Martinez-Coronado A., Esbrí J.M., Moreno M.M., Higueras P. (2021). Biomonitoring of Hg0, Hg2 and Particulate Hg in a Mining Context Using Tree Barks+. Int. J. Environ. Res. Public Health.

[83] Franzaring J., Haneke J., Sannino A., Radermacher G., Schweiger A. (2024). Effects of Legacy Mining on Mercury Concentrations in Conifer Needles and Mushrooms in Northern Palatinate, Germany. Environ. Pollut..

[84] Nováková T., Navrátil T., Schütze M., Rohovec J., Matoušková Š., Hošek M., Matys Grygar T. (2022). Reconstructing Atmospheric Hg Levels near the Oldest Chemical Factory in Central Europe Using a Tree Ring Archive. Environ. Pollut..

[85] Pleijel H., Klingberg J., Nerentorp M., Broberg M.C., Nyirambangutse B., Munthe J., Wallin G. (2021). Mercury Accumulation in Leaves of Different Plant Types—The Significance of Tissue Age and Specific Leaf Area. Biogeosciences.

[86] Pleijel H., Klingberg J., Sjöman H., Wallin G. (2025). Leaf Age Affects Mercury Accumulation in Evergreen Plants. Water Air Soil. Pollut..

[87] Frank D., Fang K., Fonti P., Siegwolf R.T.W., Brooks J.R., Roden J., Saurer M. (2022). Dendrochronology: Fundamentals and Innovations. Stable Isotopes in Tree Rings.

[88] Kang H., Liu X., Zhang X., Guo J., Huang J., Ying X., Wang Y., Zhang Q., Kang S. (2024). Important Accumulated Mercury Pool in a Remote Alpine Forest and Dynamic Accumulation Revealed by Tree Rings in China’s Qilian Mountains. Sci. Total Environ..

[89] Canning C.M., Laroque C.P., Muir D. (2023). Critical Analysis of the Past, Present, and Future of Dendrochemistry: A Systematic Literature Review. Forests.

[90] Zhang L., Qian J.-L., Planas D. (1995). Mercury Concentration in Tree Rings of Black Spruce (*Picea mariana* Mill. B.S.P.) in Boreal Quebec, Canada. Water Air Soil. Pollut..

[91] Cooke C.A., Martínez-Cortizas A., Bindler R., Sexauer Gustin M. (2020). Environmental Archives of Atmospheric Hg Deposition—A Review. Sci. Total Environ..

[92] Binda G., Di Iorio A., Monticelli D. (2021). The What, How, Why, and When of Dendrochemistry: (Paleo)Environmental Information from the Chemical Analysis of Tree Rings. Sci. Total Environ..

[93] Cocozza C., Alterio E., Bachmann O., Guillong M., Sitzia T., Cherubini P. (2021). Monitoring Air Pollution Close to a Cement Plant and in a Multi-Source Industrial Area through Tree-Ring Analysis. Environ. Sci. Pollut. Res..

[94] Ballikaya P., Marshall J., Cherubini P. (2022). Can Tree-Ring Chemistry Be Used to Monitor Atmospheric Nanoparticle Contamination over Time?. Atmos. Environ..

[95] Miyahara A.A.L., Locosselli G.M. (2024). Challenges and Advances in Intra-Annual Tree-Ring Stable Isotope Research, a Systematic Review. Dendrochronologia.

[96] Scanlon T.M., Riscassi A.L., Demers J.D., Camper T.D., Lee T.R., Druckenbrod D.L. (2020). Mercury Accumulation in Tree Rings: Observed Trends in Quantity and Isotopic Composition in Shenandoah National Park, Virginia. JGR Biogeosci..

[97] Liu Y., Liu G., Wang Z., Guo Y., Yin Y., Zhang X., Cai Y., Jiang G. (2022). Understanding Foliar Accumulation of Atmospheric Hg in Terrestrial Vegetation: Progress and Challenges. Crit. Rev. Environ. Sci. Technol..

[98] Tatzber M., Fürst A. (2023). Mercury in Tree Rings Close to Emission Sources in Austria. Environ. Sci. Pollut. Res..

[99] Ghotra A., Lehnherr I., Porter T.J., Pisaric M.F.J. (2020). Tree-Ring Inferred Atmospheric Mercury Concentrations in the Mackenzie Delta (NWT, Canada) Peaked in the 1970s but Are Increasing Once More. ACS Earth Space Chem..

[100] Clackett S.P., Porter T.J., Lehnherr I. (2021). The Tree-Ring Mercury Record of Klondike Gold Mining at Bear Creek, Central Yukon. Environ. Pollut..

[101] Baroni D., Ancora S., Franzaring J., Loppi S., Monaci F. (2023). Tree-Rings Analysis to Reconstruct Atmospheric Mercury Contamination at a Historical Mining Site. Front. Plant Sci..

[102] Fornasaro S., Ciani F., Nannoni A., Morelli G., Rimondi V., Lattanzi P., Cocozza C., Fioravanti M., Costagliola P. (2023). Tree Rings Record of Long-Term Atmospheric Hg Pollution in the Monte Amiata Mining District (Central Italy): Lessons from the Past for a Better Future. Minerals.

[103] Maillard F., Girardclos O., Assad M., Zappelini C., Pérez Mena J.M., Yung L., Guyeux C., Chrétien S., Bigham G., Cosio C. (2016). Dendrochemical Assessment of Mercury Releases from a Pond and Dredged-Sediment Landfill Impacted by a Chlor-Alkali Plant. Environ. Res..

[104] Rodríguez Martín J.A., Nanos N., Miranda J.C., Carbonell G., Gil L. (2013). Volcanic Mercury in *Pinus canariensis*. Naturwissenschaften.

[105] Rodríguez Martin J.A., Gutiérrez C., Torrijos M., Nanos N. (2018). Wood and Bark of *Pinus halepensis* as Archives of Heavy Metal Pollution in the Mediterranean Region. Environ. Pollut..

[106] Schneider L., Allen K., Walker M., Morgan C., Haberle S. (2019). Using Tree Rings to Track Atmospheric Mercury Pollution in Australia: The Legacy of Mining in Tasmania. Environ. Sci. Technol..

[107] Yang Y., Yanai R.D., Montesdeoca M., Driscoll C.T. (2017). Measuring Mercury in Wood: Challenging but Important. Int. J. Environ. Anal. Chem..

[108] Yang Y., Yanai R.D., Driscoll C.T., Montesdeoca M., Smith K.T. (2018). Concentrations and Content of Mercury in Bark, Wood, and Leaves in Hardwoods and Conifers in Four Forested Sites in the Northeastern USA. PLoS ONE.

[109] Watmough S.A., Hutchinson T.C., Evans R.D. (1998). Development of Solid Calibration Standards for Trace Elemental Analyses of Tree Rings by Laser Ablation Inductively Coupled Plasma-Mass Spectrometry. Environ. Sci. Technol..

[110] Perone A., Cocozza C., Cherubini P., Bachmann O., Guillong M., Lasserre B., Marchetti M., Tognetti R. (2018). Oak Tree-Rings Record Spatial-Temporal Pollution Trends from Different Sources in Terni (Central Italy). Environ. Pollut..

[111] Gačnik J., Živković I., Horvat M. (2025). Mercury Isotopes in the Atmosphere: Synthesis, Perspectives and Analytical Considerations. TrAC Trends Anal. Chem..

[112] Yuan T., Zhang P., Song Z., Huang S., Wang X., Zhang Y. (2023). Buffering Effect of Global Vegetation on the Air-Land Exchange of Mercury: Insights from a Novel Terrestrial Mercury Model Based on CESM2-CLM5. Environ. Int..

